# Effectiveness and Safety of COVID-19 Vaccine among Pregnant Women in Real-World Studies: A Systematic Review and Meta-Analysis

**DOI:** 10.3390/vaccines10020246

**Published:** 2022-02-06

**Authors:** Yirui Ma, Jie Deng, Qiao Liu, Min Du, Min Liu, Jue Liu

**Affiliations:** 1 Department of Epidemiology and Biostatistics, School of Public Health, Peking University, No. 38, Xueyuan Road, Haidian District, Beijing 100191, China; 1810306205@pku.edu.cn (Y.M.); 1810306145@pku.edu.cn (J.D.); 1610306236@pku.edu.cn (Q.L.); 1510306111@pku.edu.cn (M.D.); 2 Institute for Global Health and Development, Peking University, No. 5, Yiheyuan Road, Haidian District, Beijing 100871, China

**Keywords:** COVID-19 vaccine, effectiveness, safety, pregnancy, systematic review, meta-analysis

## Abstract

We aimed to assess the effectiveness and safety of coronavirus disease 2019 (COVID-19) vaccines for pregnant women in real-world studies. We searched for observational studies about the effectiveness and safety of COVID-19 vaccines among vaccinated pregnant women from inception to 6 November 2021. A total of 6 studies were included. We found that vaccination prevented pregnant women from SARS-CoV-2 infection (OR = 0.50, 95% CI, 0.35–0.79) and COVID-19-related hospitalization (OR = 0.50, 95% CI, 0.31–0.82). Messenger-RNA vaccines could reduce the risk of infection in pregnant women (OR = 0.13, 95% CI, 0.03–0.57). No adverse events of COVID-19 vaccination were found on pregnant, fetal, or neonatal outcomes. Our analysis confirmed the effectiveness and safety of COVID-19 vaccines for pregnant women. Policy makers should formulate targeted strategies to improve vaccine coverage in pregnant women.

## 1. Introduction

Coronavirus disease 2019 (COVID-19), caused by severe acute respiratory syndrome coronavirus 2 (SARS-CoV-2), has spread rapidly worldwide, with a significant increase in the cases of infection. As of 22 December 2021, there have been more than 274.6 million confirmed cases of COVID-19 globally, including more than 5.4 million deaths, reported by WHO [1]. Among these cases of infection and death, many were pregnant women. According to Centers for Disease Control and Prevention (CDC), there were 152,682 total cases of pregnant women with COVID-19, and 253 deaths in the United States, from 22 January 2020 to 20 December 2021 [2]. Statistics from CDC also showed that compared to nonpregnant women, pregnant women are at an increased risk for severe outcomes from COVID-19, such as hospitalization, intensive care or requiring a ventilator or special equipment to help them breathe [3]. In addition, there is also an increased risk for preterm birth and stillbirth and a possible risk of developing other pregnancy complications among pregnant women infected with SARS-CoV-2 [4]. Thus, it is important to devote attention to the vaccination of pregnant women to protect them from SARS-CoV-2 infection and reduce their risk of severe illness during pregnancy.

Up to 21 December 2021, WHO has listed 137 COVID-19 vaccines as being in clinical development and 194 COVID-19 vaccines as being in preclinical development, and 10 vaccines are in phase 4 clinical trials [5]. Up to 22 December 2021, WHO approved 10 COVID-19 vaccines for the emergency use listing (EUL) [6]. The types of vaccines include inactivated vaccines, messenger-RNA (mRNA) vaccines, nonreplicating viral vector vaccines, and live attenuated vaccines [7]. Expectations are high for an effective preventive and safe COVID-19 vaccine. Nowadays, more and more vaccines are being approved for marketing, such as Bharat Biotech BBV152 COVAXIN vaccine, Pfizer-BioNTech (BNT162b2) COVID-19 vaccine, Moderna COVID-19 (mRNA-1273) vaccine, Janssen Ad26.COV2.S COVID-19 vaccine, Oxford/AstraZeneca (ChAdOx1-S [recombinant] vaccine) COVID-19 vaccine, Sinopharm COVID-19 vaccine, and Sinovac-CoronaVac COVID-19 vaccine, among others. COVID-19 vaccination is gradually becoming more widely available around the world. As of 21 December 2021, a total of more than 8387.7 million vaccine doses have been administered, as reported by WHO [1]. Statistics from CDC revealed that, in United States, from 14 December 2020 to 4 December 2021, the percentage of pregnant women aged 18–48 years being fully vaccinated with a COVID-19 vaccine has been increasing gradually, and the vaccination coverage rate reached 34.8%, including 17.8% vaccinated during pregnancy, 14.5% vaccinated prior to pregnancy, and 2.5% vaccinated prior to and during pregnancy [6].

Previous studies showed that COVID-19 vaccines induce immunogenicity in pregnant women against SARS-CoV-2 infection, which is similar in nonpregnant women [8,9,10,11,12,13]. In addition, some studies showed that some anti-SARS-CoV-2 immunoglobulin can be transferred to the newborn through placenta and breastmilk, so as to provide humoral immunity [8,10]. On the other hand, several studies also reported that COVID-19 vaccination during pregnancy did not lead to significant vaccine-related adverse events or adverse outcomes or obstetric, fetal, or neonatal adverse outcomes [8,9,10,11,13]. CDC confirmed that the benefits of receiving a COVID-19 vaccine outweigh any known or potential risks of vaccination during pregnancy and suggests people who are pregnant, breastfeeding, currently trying to get pregnant, or might become pregnant in the future undergo COVID-19 vaccination [13]. 

In terms of the COVID-19 vaccination strategy, WHO suggests vaccination for pregnant women only when the benefits of vaccination outweigh the potential risks [14,15,16,17,18,19,20], but current recommendations for pregnant women to be vaccinated against COVID-19 vary from country to country. For example, except for the US, Europe and the UK encourage pregnant women to be vaccinated against COVID-19 [21,22] but China does not [23]. Due to ethical and other reasons, evidence from randomized controlled trials (RCTs) on the safety and effectiveness of COVID-19 vaccines for pregnant women are scarce. Therefore, the real-world study (RWS) of the safety and effectiveness of COVID-19 vaccines for pregnant women can provide additional evidence. However, the results of several studies of pregnant women in the real world were inconsistent [8,24]. Furthermore, previous studies have found that compared with the general population, there is a higher rate of vaccination hesitation among pregnant women, mainly due to the concerns about the safety and effectiveness of vaccines for both mothers and infants [25,26]. Hence, it is urgent to carry out a systematic study and meta-analysis on the safety and effectiveness of COVID-19 vaccination for pregnant women, which would provide more scientific evidence for the protection of pregnant women, a population vulnerable during the COVID-19 pandemic.

In this systematic review and meta-analysis, we aimed to evaluate the effectiveness and safety of COVID-19 vaccines among pregnant women in the real world to provide evidence for an improved vaccine strategy for pregnant women during the COVID-19 pandemic.

## 2. Methods

### 2.1. Search Strategy and Selection Criteria

We searched published studies, without language restrictions, from inception to 6 November 2021 in PubMed, Embase, and ScienceDirect using the following search terms: (pregnant OR pregnancy) AND (effectiveness OR safety) AND (COVID-19 OR coronavirus OR SARS-CoV-2) AND (vaccine OR vaccination). We used EndNoteX8.2 (Thomson Research Soft, Stanford, USA) to manage records, screen, and exclude duplicates. This study was strictly performed according to the Preferred Reporting Items for Systematic Reviews and Meta-Analyses (PRISMA). This study was registered on PROSPERO (CRD42021289924).

We included observational studies that examined the effectiveness and safety of COVID-19 vaccines among the pregnant women vaccinated with COVID-19 vaccine. The following studies were excluded: (1) irrelevant to the subject of the meta-analysis, such as studies that did not use COVID-19 vaccination as the exposure; (2) insufficient data to calculate the rate and outcomes for the effectiveness and safety of COVID-19 vaccines among the pregnant women; (3) duplicate studies or overlapping participants; (4) reviews, editorials, conference papers, case reports or animal experiments; and (5) studies that did not clarify the identification of COVID-19.

Studies were independently identified by two investigators (DJ and MYR) following the above criteria, and discrepancies were solved by consensus or with a third investigator (LQ).

### 2.2. Data Extraction

The primary outcome was the effectiveness of COVID-19 vaccines (including the BNT162b2 vaccine, mRNA-1273 vaccine, and adenovirus vector vaccine). The following data were extracted from the selected studies: (1) basic information of the studies, including first author, publication year, and study design; (2) characteristics of the study population, including sample sizes, age groups, trimester of pregnancy, and locations; (3) types of COVID-19 vaccines, the number of doses, the efficacy of vaccine; (4) outcomes for the effectiveness of COVID-19 vaccines: the number of SARS-CoV-2 infections, hospitalization for COVID-19, admission to the ICU for COVID-19, COVID-19-related death; and (5) outcomes for the safety of COVID-19 vaccines: the number and types of adverse pregnant, fetal or neonatal outcomes after vaccination. 

Data extraction was conducted by two investigators (DJ and MYR) independently following the criteria above, and discrepancies were solved by consensus or with a third investigator (LQ).

### 2.3. Quality Assessment

We evaluated the risk of bias using the Newcastle–Ottawa quality assessment scale for cohort studies. Cohort studies were classified as having low (≥7 stars), moderate (5–6 stars), and high risk of bias (≤4 stars) with an overall quality score of 9 stars.

Quality assessment was independently conducted by two investigators (DJ and MYR), and discrepancies were solved by consensus or with a third investigator (LQ).

### 2.4. Data Synthesis and Statistical Analysis

We performed a meta-analysis with pooled data from cohort studies and assessed the effectiveness and safety of COVID-19 vaccines by clinical outcomes (incidence of the SARS-CoV-2 infection, COVID-19-related hospitalization, severe illness, admission to the ICU, death, and adverse pregnant, fetal, or neonatal outcomes after vaccination). Random-effects or fixed-effects models were used to pool the rates and adjusted estimates across studies separately based on the heterogeneity between estimates (I²). Fixed-effects models would be used if I² ≤ 50%, which represents low to moderate heterogeneity and random-effects models would be used and tau-square would be estimated using the DerSimonian–Laird method if I² ≥ 50%, representing substantial heterogeneity. We analyzed data using Stata version 16.0 (Stata Corp, College Station, TX, USA).

## 3. Results

### 3.1. Basic Characteristics

**Figure 1:**
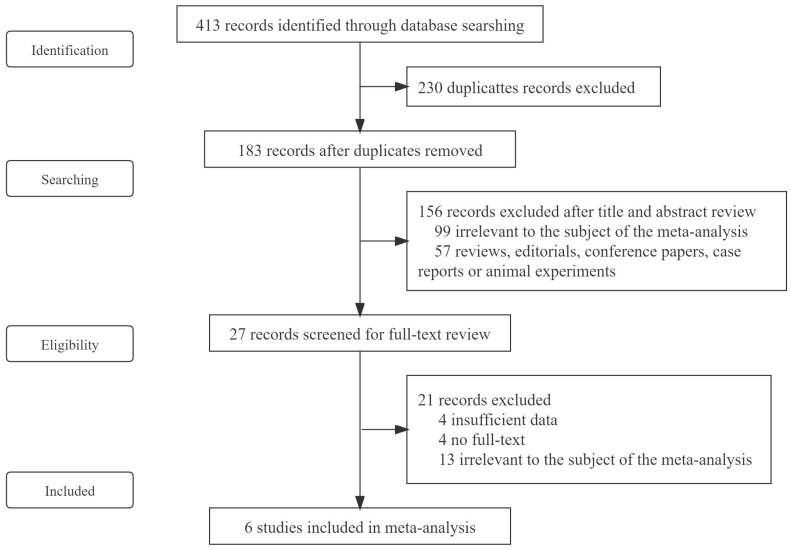
Flowchart of the study selection.

In the initial literature research, 413 potential articles were identified up to 6 November 2021 (145 in PubMed, 126 in Embase, 142 in Web of Science). A total of 230 duplicates were excluded. After reading the titles and abstracts, 156 articles were excluded based on the inclusion and exclusion criteria. Among the 27 studies under full-text review, 21 studies were excluded. Eventually, 6 studies were included in this meta-analysis based on the inclusion criteria [27,28,29,30,31,32]. The literature retrieval flow chart is shown in Figure 1. 

The included studies were observational cohort studies that involved 40,978 pregnant women (19,108 vaccinated and 21,870 unvaccinated). The basic characteristics of the included studies are shown in Table 1, Table 2, Table 3 and Table 4.

### 3.2. Effectiveness of COVID-19 Vaccines among Pregnant Women

**Figure 2:**
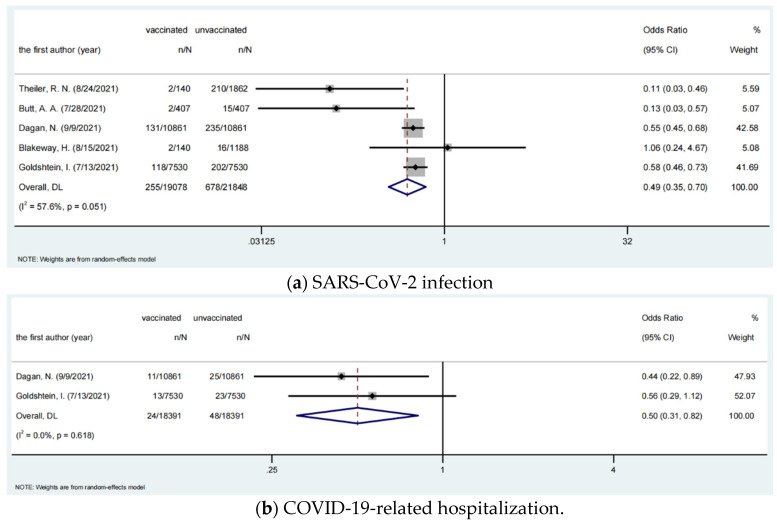
SARS-CoV-2 infection and COVID-19-related hospitalization incidence among pregnant women.

Five studies were assessed for the risk of infection after the injection of COVID-19 vaccines, involving 19,078 vaccinated pregnant women and 21,848 unvaccinated pregnant women. The pooled OR was 0.50 (95% CI, 0.35–0.70), which showed that the vaccines were protective against SARS-CoV-2 infection for the pregnant women. Two studies assessed the risk of hospital admission, involving 18,391 vaccinated pregnant women and 18,391 unvaccinated pregnant women. The pooled OR was 0.50 (95% CI, 0.31–0.82), which showed that the vaccine was protective for the pregnant women in terms of hospitalization. The risk of ICU admission and death were not assessable due to the low number of studies and insufficient data. However, we noticed that there were no deaths reported among the pregnant women in the included studies, regardless of whether they were vaccinated or unvaccinated. The results for analysis of COVID-19 vaccine effectiveness among pregnant women are shown in Table 5 and Figure 2.

### 3.3. Subgroup Analysis

We conducted subgroup analysis by location (USA, Qatar, Israel, and England), type of vaccine (BNT162b2 vaccine; 2 types of vaccines, BNT162b2 vaccine, and Moderna vaccine; as well as 3 types of vaccines, BNT162b2 vaccine, Moderna vaccine, and adenovirus vector vaccine), number of doses (≥1 and 2), population size (<1000, 1000–10,000, and >10,000), study design (prospective cohort study and retrospective cohort study), and median age of vaccinated pregnant women (<35 and ≥35). 

The reduction of SARS-CoV-2 infection among pregnant women was found in three location groups, including USA (OR = 0.14, 95% CI, 0.03–0.46), Qatar (OR = 0.13, 95% CI, 0.03–0.57), and Israel (OR = 0.56, 95% CI, 0.48–0.66), BNT162b2 (OR = 0.56, 95% CI, 0.48–0.66), BNT162b2 and Moderna (OR = 0.13, 95% CI, 0.03–0.57) vaccine groups, 1000–10,000 (OR = 0.14, 95% CI, 0.03–0.46), and >10,000 (OR = 0.56, 95% CI, 0.48–0.66) population size groups, prospective cohort (OR = 0.54, 95% CI, 0.41–0.77) and retrospective cohort (OR = 0.13, 95% CI, 0.03–0.57) study design groups, and <35 y median age group (OR = 0.47, 95% CI, 0.33–0.68) (Table 6).

### 3.4. Safety of COVID-19 Vaccines among Pregnant Women

We assessed the risk of adverse pregnancy outcomes among vaccinated and unvaccinated pregnant women. A total of 2 studies involving 140 vaccinated pregnant women and 1862 unvaccinated pregnant women were assessed for the risk of uterine rupture (OR = 4.42, 95% CI, 0.18–108.91), third- or fourth-degree laceration (OR = 0.72, 95% CI, 0.17–3.00), return to the operating room within 72 h of delivery (OR = 2.23, 95% CI, 0.27–18.62), 5 min Apgar score < 7 (OR = 1.05, 95% CI, 0.32–3.45), quantitative blood loss > 1000 mL (OR = 1.42, 95% CI, 0.60–3.35), transfusion (OR = 1.46, 95% CI, 0.93–2.30), pregestational hypertension (OR = 1.14, 95% CI, 0.69–1.89). A total of 2 studies involving 129 vaccinated pregnant women and 1580 unvaccinated pregnant women were assessed for the risk of thromboembolism within 4 weeks before or after delivery (OR = 2.44, 95% CI, 0.12–51.05). A total of 2 studies involving 129 vaccinated pregnant women and 1581 unvaccinated pregnant women were assessed for the risk of stroke within 4 weeks before or after delivery (OR = 2.44, 95% CI, 0.12–51.08). A total of 3 studies involving 273 vaccinated pregnant women and 2261 unvaccinated pregnant women were assessed for the risk of postpartum hemorrhage with blood transfusion (OR = 1.18, 95% CI, 0.63–2.23), spontaneous vaginal delivery (OR = 0.90, 95% CI, 0.69–1.17), operative vaginal delivery (OR = 1.51, 95% CI, 0.96–2.40), cesarean delivery (OR = 0.80, 95% CI, 0.74–1.29). A total of 3 studies involving 7670 vaccinated pregnant women and 9392 unvaccinated pregnant women were assessed for the risk of eclampsia or preeclampsia up to 72 h from delivery (OR = 0.91, 95% CI, 0.51–1.64). We found that there was not a direct association between vaccination and these adverse pregnancy outcomes in vaccinated pregnant women. The analysis results of adverse pregnancy outcomes among pregnant women with COVID-19 vaccination are shown in Table 7.

**Figure 3:**
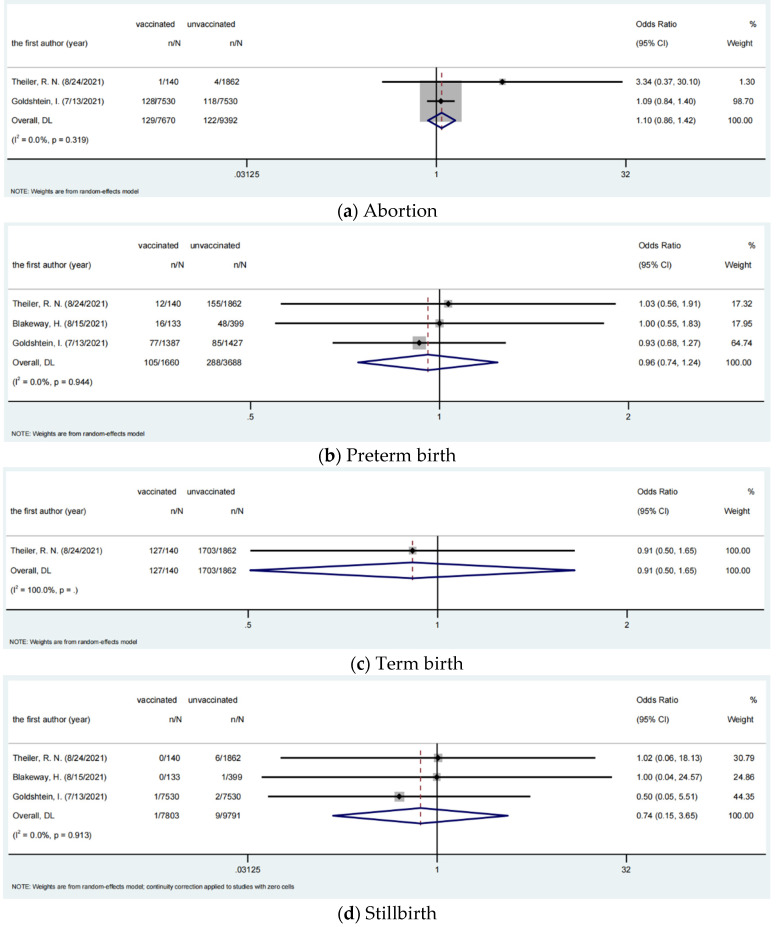
Fetal outcomes among pregnant women.

We assessed the risk of 4 fetal outcomes among vaccinated and unvaccinated pregnant women, including 3 studies for abortion, 4 studies for preterm birth, 2 studies for term birth, and 4 studies for stillbirth. The pooled OR were 1.10 (95% CI, 0.86–1.42), 0.96 (95% CI, 0.74–1.24), 0.91 (95% CI, 0.50–1.65), and 0.74 (95% CI, 0.15–3.65), respectively, which showed there were no significant differences in fetal outcomes between vaccinated and unvaccinated pregnant women. The analysis results of adverse fetal outcomes among pregnant women with COVID-19 vaccination are shown in Table 8 and Figure 3.

We assessed the risk of adverse neonatal outcomes among vaccinated and unvaccinated pregnant women. A total of 2 studies involving 140 vaccinated pregnant women and 1862 unvaccinated pregnant women were assessed for the risk of low or very low birthweight (<2500 g) (OR = 1.35, 95% CI, 0.76–2.40), neonatal birth trauma (OR = 0.57, 95% CI, 0.03–9.77), hypoxic–ischemic encephalopathy (OR = 4.42, 95% CI, 0.18–108.91). A total of 3 studies involving 273 vaccinated pregnant women and 2261 unvaccinated pregnant women were assessed the risk of admission to the neonatal ICU (OR = 1.08, 95% CI, 0.48–2.42). We did not find any neonatal deaths among vaccinated and unvaccinated pregnant women. The results suggest that there is not an obvious connection between vaccination and adverse neonatal outcomes among vaccinated pregnant women. The analysis results of adverse neonatal outcomes among pregnant women with COVID-19 vaccination are shown in Table 9.

### 3.5. Quality Evaluation and Publication Bias

We evaluated the quality of the included articles according to the Newcastle–Ottawa quality assessment scale, and all the included articles were of good quality (≥7 stars). 

## 4. Discussion

To our knowledge, this is the first systematic review and meta-analysis assessing effectiveness and safety of COVID-19 vaccine among pregnant women based on real-world studies that reports the incidence of clinical status of COVID-19 and adverse events on pregnant, fetal, or neonatal outcomes. This meta-analysis consisted of a total of 6 observational studies involving 40,978 pregnant women. We found that COVID-19 vaccination caused the risk of SARS-CoV-2 infection and COVID-19-related hospitalization to both decrease by 50%. Moreover, no adverse events of COVID-19 vaccination were found on pregnant, fetal, or neonatal outcomes. Nowadays, data about the safety and effectiveness of vaccines given to pregnant women has been growing. These data suggest that the benefits of vaccination to the pregnant women outweigh the known or potential risks, and some regions or countries such as the USA, Europe, the UK, Australia, Canada, and France have adjusted their strategies to encourage pregnant women to get vaccinated against COIVD-19 [21,22,33,34,35,36]. Hence, our results confirm the effectiveness and safety of COVID-19 vaccine for pregnant women, which can allay pregnant women’s fears about the uncertainty of COVID-19 vaccines and serve as a reference for the relevant departments to formulate policies to improve the acceptance of vaccines among pregnant women.

Analysis of the available data shows that vaccination with COVID-19 mRNA vaccines (including BNT162b2 and Moderna vaccines) reduces the risk of SARS-CoV-2 infection in pregnant women (OR = 0.13, 95% CI, 0.03–0.57). The BNT162b2 vaccine alone also decreased the risk of SARS-CoV-2 infection (OR = 0.56, 95% CI, 0.48–0.66). The BNT162b2 and Moderna vaccines are mRNA vaccines encapsulated in lipid nanoparticles, which encode the viral spike protein of SARS-CoV-2. Both are given in two doses. BNT162b2 is recommended for individuals aged 12 and above, and Moderna is recommended for those aged 18 and above. Previous studies have shown that these two vaccines reduce the risk of SARS-CoV-2 infection in nonpregnant people, and the efficacy of both vaccines is ≥90%, which is consistent across age, sex, race, and ethnicity, and results regarding their safety are reassuring [37,38,39,40]. In addition, a meta-analysis showed that two doses of COVID-19 vaccine were 85% or more effective in preventing any clinical status of COVID-19 in general population [41]. Similar results were observed in pregnant women. Dagan found that within 7–56 days of the second dose of BNT162b2 vaccine for pregnant women aged ≥16, the vaccine was 96% effective against recorded infections and 97% effective against symptomatic infections, and that the double-dose vaccine was more effective for pregnant women than the single dose [30].

This is related to the level of antibodies in the serum of pregnant women after vaccination. Gray quantified SARS-CoV-2 spike and receptor-binding domain (RBD) immunoglobulin G (IgG), immunoglobulin A (IgA), and immunoglobulin M (IgM) in 131 vaccine recipients of child-bearing age. He found vaccine-induced antibody titers were comparable to those of nonpregnant women during pregnancy and lactation (pregnant: median 5.59 and interquartile range 4.68–5.89; lactating: median 5.74 and interquartile range 5.06–6.22; nonpregnant: median 5.62 and interquartile range 4.77–5.98). Besides, all titers caused by vaccine were significantly higher than those caused by natural infection of SARS-CoV-2 in pregnant women [42]. IgM, IgG, and IgA levels rose significantly in pregnant women after receiving the first dose of BNT162B2 vaccine, and IgG levels further increased after the second injection. Two weeks after the second dose, the main antibody response in the serum of pregnant, lactating, and nonpregnant women was dominated by IgG [42,43].

After COVID-19 vaccination, the vaccine’s protection and ability to prevent infection with new strains may decrease over time. Timely booster shots can allow production of neutralizing antibodies that have been gradually reduced grow rapidly or rebound to produce better results. At present, various countries differ with respect to recommendations to get a booster shot. Israel, China, the UK, and the EU have all actively recommended booster vaccination for priority groups (such as elderly people with low immunity, high-risk groups of SARS-CoV-2 infection, etc.) [44,45,46]. The US recommends that those aged 16 or above get a booster shot [47]. However, WHO believes that there is insufficient evidence to justify the need for widespread booster vaccination and, instead, focus should be on improving global primary vaccination coverage to alleviate serious inequities in vaccine distribution [48]. Therefore, it is important to determine whether pregnant women are regarded as a priority group for booster vaccination. There is currently insufficient evidence to support the need for booster vaccination in pregnant women. 

We found a lower risk of COVID-19-related infections in younger age groups. Studies have shown that advanced maternal age (≥35 years) is an independent risk factor for pregnancy complications, delivery complications, and adverse pregnancy outcomes [49]. To our knowledge, the immune system plays an important role in defending against infections, though previous studies have found that aging influences immune response to coronavirus infection. For instance, immunosenescence can inhibit adaptive immunity mediated by T and B cells, with increasing susceptibility to infection and clinical outcomes thereafter [50]. In addition, the literature has shown that pregnancy is a special state of immune tolerance in which cell-mediated immunity is attenuated through a range of mechanisms to prevent fetal rejection during pregnancy, thus leading to unique susceptibility to infectious disease pandemics and increased severity of virus-related diseases. As a result, infectious diseases such as COVID-19 make pregnant women vulnerable to adverse pregnancy outcomes, not only increasing the risk of maternal and perinatal morbidity but even leading to maternal and perinatal death [12,51,52,53]. A systematic review based on 63 observational studies reveals that maternal age in combination with other factors can predict adverse pregnancy outcomes associated with COVID-19, and that older women infected with SARS-CoV-2 during the third trimester of pregnancy may have the highest rates of morbidity and mortality [54]. In our study, we did not observe a higher risk SARS-CoV-2 infection in older age groups, but aging remains a suspected risk factor for pregnant women. However, we were not able to assess the risk of COVID-19-related hospitalizations in the older age group due to insufficient data. Moreover, we did not have data available to determine accurately whether the hospitalization was due to SARS-CoV-2 infection or COVID-19-related factors combined with pregnancy-related indicators. Therefore, studies about the impact of maternal age on the safety and effectiveness of vaccination as well as the correlation between pregnancy, age, and immunization should be conducted in the future.

In the subgroup analysis, among the pregnant women, we have found the protection of SARS-CoV-2 infection in retrospective cohort studies as well as the protection of SARS-CoV-2 infection in prospective cohort studies. Because there are so few data, we cannot assess the impact of prospective studies on COVID-19-related hospitalization. Retrospective and prospective studies are both observational cohort studies. The difference lies in that the former are collated and analyzed based on previous data, while the latter are carried out by researchers according to the requirements of topic selection and design. Both assume that researchers will follow the population over time, measure several major and minor exposure factors and outcomes, and evaluate the correlation between multiple exposures and multiple outcomes, both with high accuracy and efficiency [55,56,57]. A study testing the comparability between retrospective life history data and prospective birth cohort study data showed a high degree of similarity [58]. Therefore, no statistic difference in vaccine effectiveness was found between the two cohort analyses in our analysis.

In the subgroup analysis, the group of England did not show any difference as for other location groups, which may be due to differences in the risk of infection between different countries. Although some kind of interaction with regard to vaccine effectiveness could be expected in different countries, their safety characteristics should be the same [24]. CDC has summarized growing evidence from countries and regions about the safety and effectiveness of COVID-19 vaccination during pregnancy: First, COVID-19 vaccines do not cause COVID-19 infection, including in people who are pregnant or their babies. Second, early data on the safety of receiving an mRNA COVID-19 vaccine (Moderna or Pfizer-BioNTech) during pregnancy are reassuring. Third, early data suggest receiving an mRNA COVID-19 vaccine during pregnancy reduces the risk of infection. Fourth, vaccination during pregnancy leads to the production of antibodies that might protect the baby. Fifth, no safety concerns were found in animal studies [59]. Consistent with previous studies [24,60,61], we found no adverse effects of COVID-19 vaccination on pregnancy-related, fetal, or neonatal outcomes. In addition, studies have found that Pfizer vaccine can induce effective maternal to neonatal transfer of IgG [29,43,62]. The longer the time interval from vaccination to delivery, the higher the maternal and neonatal antibody levels and the cord-to-maternal ratio [63,64]. After the receipt of the second vaccine dose, the maternal and neonatal antibody levels change by −10.9% and −11.7% per week [63]. This suggests that maternal vaccination protects infants. 

Our study has some limitations. First, the population we included was limited to a few specific regions or countries, and the effectiveness and efficacy of vaccines for pregnant women worldwide cannot be completely and accurately assessed. Second, the vaccination status we included was limited to 2 doses and ≥1 dose, which overlapped, and there was no evaluation of the effectiveness of COVID-19 vaccine after the first injection in pregnant women. Third, due to the limited available data and the low hospitalization admission threshold for pregnant women, we could not precisely determine whether these hospitalizations were due to SARS-CoV-2 infection or COVID-19 combined with pregnancy indicators. Fourth, we evaluated the publication bias based on Harbord’s modified test, and *p* values for the meta-analysis were all more than 0.05; however, they were limited for potential publication as the number of studies was too small. Fifth, the DerSimonian–Laird method we used to combine the effect size and assess the heterogeneity might underestimate the uncertainty due to the few numbers of studies involved the meta-analysis. In addition, we did not adopt the Hartung–Knapp method to adjust our analysis results, which may cause the potential risk of underestimating the between-study variability and even the risk of false positive results. Sixth, the results of the subgroup analysis for hospitalization were not informative as this outcome was evaluated for just two studies, so we removed them. Seventh, the mRNA + adenovirus vector vaccine group did not show differences as the data source studies focused on the effect of vaccination, not vaccine type, on the outcomes of pregnant women, which causes difficulties for a separate discussion of the effectiveness of adenovirus vector vaccine.

## 5. Conclusions

Our results suggest that COVID-19 vaccines are effective in reducing the incidence of SARS-CoV-2 infection and COVID-19-related hospitalization among pregnant women. No adverse effects of COVID-19 vaccination were found on pregnant, fetal, or neonatal outcomes. Our results confirm the effectiveness and safety of COVID-19 vaccination for pregnant women. Our findings can serve as a reference for relevant policy makers to formulate targeted strategies to improve the COVID-19-related vaccine policy for pregnant women. Moreover, reducing hesitancy regarding COVID-19 vaccines is also helpful for improving vaccination coverage and protect pregnant women from SARS-CoV-2 infection toward ending the pandemic.

## Figures and Tables

**Table 1:** Findings of included original studies for vaccine effectiveness.

Record Number	First Author	Published Time	Study Design	Location	Vaccine Type	No. of Dose	Median Age	Median Gestational Age	Vaccinated Pregnant Women with SARS-CoV-2 Infection (n/N)	Unvaccinated Pregnant Women with SARS-CoV-2 Infection (n/N)	Vaccinated Pregnant Women with Hospitalization (n/N)	Unvaccinated Pregnant Women with Hospitalization (n/N)	Vaccinated Pregnant Women with Severe Illness or ICU Admission (n/N)	Unvaccinated Pregnant Women with Severe Illness or ICU Admission (n/N)	Vaccinated Pregnant Women with Death (n/N)	Unvaccinated Pregnant Women with Death (n/N)	Risk of Bias
1	Theiler, R. N.	2021.8	Retrospective cohort study	USA	BNT162b2+Moderna+ adenovirus vector vaccine	≥1	31.8	Last trimester	2/140	210/2862	-	-	1/140	2/1862	0/140	0/1862	Low
2	Butt, A. A.	2021.7	Prospective cohort study	Qatar	BNT162b2+Moderna	2	32	Early trimester	2/407	15/407	-	-	-	-	0/407	0/407	Low
3	Collier, A. Y.	2021.5	Prospective cohort study	Israel	BNT162b2+Moderna	2	35	Second trimester	-	-	0/30	0/22	0/30	0/22	0/30	0/22	Low
4	Dagan, N.	2021.9	Retrospective cohort study	Israel	BNT162b2	2	30	-	131/10,861	235/10,861	11/18,061	25/18,061	0/10,861	4/10,861	0/10,861	0/10,861	Low
5	Blakeway, H.	2021.8	Retrospective cohort study	England	BNT162b2+Moderna+adenovirus vector vaccine	≥1	35	Last trimester	2/140	16/1188	-	-	8/133	16/399	-	-	Low
6	Goldshtein, I.	2021.7	Retrospective cohort study	Israel	BNT162b2	≥1	31.1	-	118/7530	202/7530	13/7530	23/7530	-	-	0/7530	0/7530	Low

**Table 2:** Findings of included original studies for adverse pregnancy outcomes.

**Record Number**	**First Author**	**Published Time**	**Vaccinated Pregnant Women with Uterine Rupture**	**Unvaccinated Pregnant Women with Uterine Rupture**	**Vaccinated Pregnant Women with Third- or Fourth-Degree Laceration**	**Unvaccinated Pregnant Women with Third- or Fourth-Degree Laceration**	**Vaccinated Pregnant Women with Return to the Operating Room within 72 h of Delivery**	**Unvaccinated Pregnant Women with Return to the Operating Room within 72 h of Delivery**	**Vaccinated Pregnant Women with 5 min Apgar Score < 7**	**Unvaccinated PREGNANT Women with 5 min Apgar Score < 7**	**Vaccinated Pregnant Women with Quantitative Blood Loss >1000 mL**	**Unvaccinated Pregnant Women with Quantitative Blood Loss >1000 mL**	**Vaccinated Pregnant Women with Transfusion**	**Unvaccinated Pregnant Women with Transfusion**	**Vaccinated Pregnant Women with postpartum Hemorrhage**	**Unvaccinated Pregnant Women with Postpartum Hemorrhage**	**Risk of Bias**
			**(n/N)**	**(n/N)**	**(n/N)**	**(n/N)**	**(n/N)**	**(n/N)**	**(n/N)**	**(n/N)**	**(n/N)**	**(n/N)**	**(n/N)**	**(n/N)**	**(n/N)**	**(n/N)**	
1	Theiler, R. N.	2021.8	0/140	1/1862	2/140	37/1862	1/140	6/1862	3/140	38/1862	6/140	57/1862	25/140	241/1862	1/140	5/1862	low
2	Butt, A. A.	2021.7	-	-	-	-	-	-	-	-	-	-	-	-	-	-	low
3	Collier, A. Y.	2021.5	0/30	0/22	0/30	0/22	0/30	0/22	0/30	0/22	0/30	0/22	0/30	0/22	0/30	0/22	low
4	Dagan, N.	2021.9	-	-	-	-	-	-	-	-	-	-	-	-	-	-	low
5	Blakeway, H.	2021.8	-	-	-	-	-	-	-	-	-	-	-	-	13/133	36/399	low
6	Goldshtein, I.	2021.7	-	-	-	-	-	-	-	-	-	-	-	-	-	-	low
**Record Number**	**First Author**	**Published Time**	**Vaccinated Pregnant Women with Spontaneous Vaginal Delivery**	**Unvaccinated Pregnant Women with Spontaneous Vaginal Delivery**	**Vaccinated Pregnant Women with Operative Vaginal Delivery**	**Unvaccinated Pregnant Women with Operative Vaginal Delivery**	**Vaccinated Pregnant Women with Cesarean Delivery**	**Unvaccinated Pregnant Women with Cesarean Delivery**	**Vaccinated Pregnant Women with Eclampsia or Preeclampsia**	**Unvaccinated Pregnant Women with Eclampsia or Preeclampsia**	**Vaccinated Pregnant Women with Pregestational Hypertension**	**Unvaccinated Pregnant Women with Pregestational Hypertension**	**Vaccinated Pregnant Women with Thromboembolism**	**Unvaccinated Pregnant Women with Thromboembolism**	**Vaccinated Pregnant Women with Stroke**	**Unvaccinated Pregnant Women with Stroke**	**Risk of Bias**
			**(n/N)**	**(n/N)**	**(n/N)**	**(n/N)**	**(n/N)**	**(n/N)**	**(n/N)**	**(n/N)**	**(n/N)**	**(n/N)**	**(n/N)**	**(n/N)**	**(n/N)**	**(n/N)**	
1	Theiler, R. N.	2021.8	89/140	1238/1862	7/140	69/1862	44/140	555/1862	1/140	23/1862	19/140	225/1862	0/129	2/1580	0/129	2/1581	low
2	Butt, A. A.	2021.7	-	-	-	-	-	-	-	-	-	-	-	-	-	-	low
3	Collier, A. Y.	2021.5	0/30	0/22	0/30	0/22	0/30	0/22	0/30	0/22	0/30	0/22	0/30	0/22	0/30	0/22	low
4	Dagan, N.	2021.9	-	-	-	-	-	-	-	-	-	-	-	-	-	-	low
5	Blakeway, H.	2021.8	71/133	221/399	21/133	42/399	41/133	136/399	-	-	-	-	-	-	-	-	low
6	Goldshtein, I.	2021.7	-	-	-	-	-	-	20/7530	21/7530	-	-	-	-	-	-	low

**Table 3:** Findings of included original studies for fetal outcomes.

Record Number	First Author	Published Time	Vaccinated Pregnant Women with Abortion (n/N)	Unvaccinated Pregnant Women with Abortion (n/N)	Vaccinated Pregnant Women with Preterm Birth (n/N)	Unvaccinated Pregnant Women with Preterm Birth (n/N)	Vaccinated Pregnant Women with Term Birth (n/N)	Unvaccinated Pregnant Women with Term Birth (n/N)	Vaccinated Pregnant Women with Stillbirth (n/N)	Unvaccinated Pregnant Women with Stillbirth (n/N)	Risk of Bias
1	Theiler, R. N.	2021.8	1/140	4/1862	12/140	155/1862	127/140	1703/1862	0/140	6/1862	Low
2	Butt, A. A.	2021.7	-	-	-	-	-	-	-	-	Low
3	Collier, A. Y.	2021.5	0/30	0/22	0/30	0/22	0/30	0/22	0/30	0/22	Low
4	Dagan, N.	2021.9	-	-	-	-	-	-	-	-	Low
5	Blakeway, H.	2021.8	-	-	16/133	48/399	-	-	0/133	1/399	Low
6	Goldshtein, I.	2021.7	128/7530	118/7530	77/1387	85/1427	-	-	1/7530	2/7530	Low

**Table 4:** Findings of included original studies for adverse neonatal outcomes.

Record Number	First Author	Published Time	Vaccinated Pregnant Women with (Very) Low Birthweight (<2500 g) (n/N)	Unvaccinated Pregnant Women with (Very) Low Birthweight (<2500 g) (n/N)	Vaccinated Pregnant Women with Neonatal Birth Trauma (n/N)	Unvaccinated Pregnant Women with Neonatal Birth Trauma (n/N)	Vaccinated Pregnant Women with Admission to the Neonatal ICU (n/N)	Unvaccinated Pregnant Women with Admission to the Neonatal ICU (n/N)	Vaccinated Pregnant Women with Neonatal Death (n/N)	Unvaccinated Pregnant Women with Neonatal Death (n/N)	Vaccinated Pregnant Women with Neonatal Hypoxic–Ischemic Encephalopathy(n/N)	Unvaccinated Pregnant Women with Neonatal Hypoxic–Ischemic Encephalopathy(n/N)	Risk of Bias
1	Theiler, R. N.	2021.8	14/140	142/1862	0/140	11/1862	1/140	11/1862	0/140	0/1862	0/140	1/1862	low
2	Butt, A. A.	2021.7	-	-	-	-	-	-	-	-	-	-	low
3	Collier, A. Y.	2021.5	0/30	0/22	0/30	0/22	0/30	0/22	0/30	0/22	0/30	0/22	low
4	Dagan, N.	2021.9	-	-	-	-	-	-	-	-	-	-	low
5	Blakeway, H.	2021.8	-	-	-	-	7/133	20/399	-	-	-	-	low
6	Goldshtein, I.	2021.7	-	-	-	-	-	-	-	-	-	-	low

**Table 5:** Effectiveness of COVID-19 vaccine among pregnant women.

Outcomes	Vaccinated Pregnant Women n/N	Unvaccinated Pregnant Women n/N	OR	95%CI	*p*-Value	I^2^%	P-Heterogeneity
Infection	255/19,078	678/21,848	0.495	0.348–0.703	0.000	57.6	0.051
Hospitalization	24/18,391	48/18,391	0.501	0.306–0.818	0.006	0	0.618
Death	0	0	-	-	-	-	-

Death: no studies found with sufficient data to be analyzed.

**Table 6:** Subgroup analysis of incidence of infection after COVID-19 vaccination, by location, vaccine type, number of doses, population size, study design and median age of participants.

Location	OR	95%CI	Weight%	*p*-Value	I2	P
USA	0.114	0.028–0.464	5.59			
Qatar	0.129	0.029–0.568	5.07			
Israel	0.564	0.482–0.660	84.27			
England	1.062	0.242–4.666	5.08			
Vaccine type	OR	95%CI	weight%	*p*-value	I2	P
BNT162b2	0.564	0.482–0.660	84.27			
BNT162b2+Moderna	0.129	0.029–0.568	5.07			
mRNA + adenovirus vector vaccine	0.343	0.039–3.057	10.66			
No. Of dose	OR	95%CI	weight%	*p*-value	I2	P
≥1	0.441	0.160–1.214	52.35			
2	0.324	0.082–1.278	47.65			
Population size	OR	95%CI	weight%	*p*-value	I2	P
<1000	0.370	0.047–2.920	10.14			
1000–10,000	0.114	0.028–0.464	5.59			
>10,000	0.564	0.482–0.660	84.27			
Study design	OR	95%CI	weight%	*p*-value	I2	P
Retrospective cohort study	0.542	0.408–0.722	94.93			
Prospective cohort study	0.129	0.029–0.568	5.07			
Median age	OR	95%CI	weight%	*p*-value	I2	P
<35	0.471	0.325–0.682	94.92			
≥35	1.062	0.242–4.666	5.08			
Overall	0.495	0.348–0.703	100.00	<0.05	57.6%	0.051

**Table 7:** Adverse pregnancy outcomes among pregnant women with and without COVID-19 vaccination.

Outcomes	Source of Data	Vaccinated Pregnant Women n/N	Unvaccinated Pregnant Women n/N	OR	95%CI	*p*-Value	I^2^	P-Heterogeneity
Uterine rupture	1, 3	0/140	1/1862	4.416	0.179–108.909	0.364	-	-
Third- or fourth-degree laceration	1, 3	2/140	37/1862	0.715	0.170–2.997	0.646	-	-
Return to the operating room within 72 h of delivery	1, 3	1/140	6/1862	2.225	0.266–18.615	0.460	-	-
5 min Apgar score < 7	1, 3	3/140	38/1862	1.051	0.320–3.449	0.934	-	-
Quantitative blood loss > 1000 mL	1, 3	6/140	57/1862	1.418	0.600–3.348	0.426	-	-
Transfusion	1, 3	25/140	241/1862	1.462	0.929–2.300	0.100	-	-
Postpartum hemorrhage with blood transfusion	1, 3, 5	14/273	41/2261	1.181	0.625–2.234	0.608	0	0.437
Spontaneous vaginal delivery	1, 3, 5	160/273	1459/2261	0.899	0.690–1.171	0.429	0	0.861
Operative vaginal delivery	1, 3, 5	28/273	111/2261	1.514	0.955–2.401	0.078	0	0.759
Cesarean delivery	1, 3, 5	85/273	691/2261	0.979	0.741–1.293	0.880	0	0.432
Eclampsia or preeclampsia up to 72 h from delivery	1, 3, 6	21/7670	44/9392	0.912	0.507–1.640	0.759	0	0.638
Gestational hypertension	1, 3	19/140	225/2862	1.142	0.691–1.890	0.604	-	-
Thromboembolism within 4 weeks before or after delivery	1, 3	0/129	2/1580	2.438	0.116–51.047	0.566	-	-
Stroke within 4 weeks before or after delivery	1, 3	0/129	2/2581	2.439	0.116–51.080	0.566	-	-

**Table 8:** Adverse fetal outcomes among pregnant women with and without COVID-19 vaccination.

Outcomes	Vaccinated Pregnant Women n/N	Unvaccinated Pregnant Women n/N	OR	95%CI	*p*-Value	I^2^	P-Heterogeneity
Abortion	129/7670	122/9392	1.102	0.858–1.416	0.447	0	0.319
Preterm birth	105/1660	288/3688	0.958	0.742–1.237	0.743	0	0.944
Term birth	127/140	1703/1862	0.912	0.504–1.651	0.761	-	-
Stillbirth	1/7803	9/9791	0.738	0.149–3.651	0.710	0	0.913

**Table 9:** Adverse neonatal outcomes among pregnant women with and without COVID-19 vaccination.

Outcomes	Source of Data	Vaccinated Pregnant Women n/N	Unvaccinated Pregnant Women n/N	OR	95%CI	*p*-Value	I^2^	P-Heterogeneity
Neonatal birth trauma	1, 3	0/140	11/1862	0.573	0.034–9.773	0.700	-	-
(Very) low birthweight (<2500 g)	1, 3	14/140	142/1862	1.346	0.755–2.399	0.314	-	-
Admission to the neonatal ICU	1, 3, 5	8/273	31/2261	1.076	0.478–2.424	0.860	0	0.903
Neonatal death	1, 3	0	0	-	-		-	-
Neonatal hypoxic–ischemic encephalopathy	1, 3	0/140	1/1862	4.416	0.179–108.909	0.364	-	-

Death: no studies found with sufficient data to be analyzed.

## Data Availability

Data are available from the corresponding author by request.
